# Laccase pretreatment of wheat straw: effects of the physicochemical characteristics and the kinetics of enzymatic hydrolysis

**DOI:** 10.1186/s13068-019-1499-3

**Published:** 2019-06-24

**Authors:** Zhichao Deng, Ao Xia, Qiang Liao, Xianqing Zhu, Yun Huang, Qian Fu

**Affiliations:** 1 Key Laboratory of Low-grade Energy Utilization Technologies and Systems, Chongqing University, Ministry of Education, Chongqing, 400044 China; 2 Institute of Engineering Thermophysics, Chongqing University, Chongqing, 400044 China

**Keywords:** Lignocellulose, Laccase treatment, Kinetic model, Saccharification, Wheat straw

## Abstract

**Background:**

Wheat straw, the most abundant lignocellulosic biomass in China, is rich in cellulose that can be hydrolyzed and then converted into biofuels, such as bioethanol and biohydrogen. However, the accessibility of cellulose and the enzyme activity are greatly reduced in the presence of lignin. This significantly increases the enzyme cost in the saccharification, which hampers industrial production of lignocellulosic biofuels. In this study, a laccase treatment system mediated by 1-hydroxybenzotriazole was employed to modify and degrade lignin to enhance subsequent enzymatic saccharification of wheat straw. A kinetic model considering enzyme adsorption on lignin was proposed to estimate the saccharification performance.

**Results:**

Fourier transform infrared spectroscopy (FTIR) analyses showed that the peak intensity of lignin structure characteristics significantly changed after laccase-mediated system (LMS) treatment. 2D-nuclear magnetic resonance (NMR) analyses indicated that the aromatic ether bonds were cleaved and that guaiacyl (G) was oxidized after LMS treatment. X-ray diffraction (XRD) analyses suggested that the crystallinity of lignocellulose increased due to the partial degradation of lignin. As a result, the nonproductive adsorption of the enzyme on lignin was reduced by 28%, while the reducing sugar yield increased by 26%. A semi-empirical kinetic model was used to estimate the reducing sugar yield, the initial hydrolysis rate (*K*_*M*_) and deactivation rate coefficient (*α*) of LMS-pretreated wheat straw were 0.157 (h^−1^) and 0.214 (h^−1^), respectively. The model showed high accuracy (predicting error < 10%) for describing the behavior of laccase-treated wheat straw hydrolysis when the solid loading is < 5%.

**Conclusions:**

The adsorption ability of the enzyme to lignin was reduced after LMS pretreatment. Physicochemical analyses showed that the chemical groups of lignin and lignocellulose were changed, with the crystallinity of the lignocellulose increasing after LMS treatment. A semi-empirical kinetic model was proposed to estimate the reducing sugar yield, which showed high accuracy for predicting the hydrolysis performance of laccase-treated wheat straw.

## Background

With the rapid increase of global energy demand in recent years, energy shortage has become a common problem in all countries. It has been predicted that the growth of energy demand will come to a peak in 2030, and as a result, the production of easily exploitable fossil fuels may not satisfy the need at that time [1]. In addition, climate change and a rise in sea levels, caused by a large amount of greenhouse gases emitted by the combustion of fossil fuels, are also a growing concern of global countries [2, 3]. The most effective solution to this situation is to aggressively develop renewable energy sources, one such example is biofuels, which have received a great deal of attention and research as a kind of renewable energy [4]. In this context, lignocellulosic biomass has become a sustainable alternative resource of biofuels, such as bioethanol and biohydrogen, since it is the most abundant biomass resource on the planet [5].

Lignocellulose is a complex three-dimensional polymer composed of cellulose, hemicellulose and lignin. Cellulose and hemicellulose are polysaccharides that can be hydrolyzed by enzymes into reducing sugars and then utilized [6]. Lignin is a water-insoluble, cross-linked aromatic polymer consisting of three phenyl-propane units linked together by ether and carbon–carbon bonds, which protects cellulose from degradation in nature and makes lignocellulose hard to utilize [7]. Lignin has a higher adsorption capacity for hydrolytic enzymes than carbohydrates, which leads to a decrease in enzyme concentration and reduction of enzyme activity, and it also covers the surface of cellulose to reduce its accessibility [8]. This significantly increases the enzyme cost in the saccharification stage of lignocellulosic biofuel production. Therefore, achieving efficient saccharification is the bottleneck in biofuel production from lignocellulosic materials [9].

Mechanical, physical and chemical pretreatment methods can effectively destroy the natural structure of lignocellulose to make it easy to hydrolyze by enzymes [10]. Nevertheless, such methods require large energy input and equipment maintenance cost. In addition, the byproducts from the pretreatment process would cause inhibition of the subsequent saccharification and fermentation processes [11]. Biological pretreatments have been widely used due to their environmental friendliness, low energy input and mild reaction conditions [12]. Biological pretreatment is based on the reaction of lignin-degrading microorganisms, and their secreted enzymes that act upon lignocellulosic biomass to improve the conversion efficiency of polysaccharides in the enzymatic hydrolysis stage [13]. As one kind of ligninolytic enzymes secreted by fungi, laccases can oxidize the phenolic polymer to produce water by using molecular oxygen as the final electron acceptor, which are most likely to achieve large-scale industrial production [14]. Although laccase can oxidize phenolic subunits, it is incapable of oxidizing non-phenolic subunits with a high redox potential in lignin. The mediator is a molecule that transmits electrons between laccase and substrates, such as non-phenols and macromolecular lignin [15]. It enables laccase to oxidize non-phenolic subunits in the lignin. Therefore, laccases are usually combined with a mediator to oxidize lignin in a lignocellulosic biomass.

A few recent literatures have proven that the LMS (laccase mediator system) is sufficiently capable of degrading lignin in lignocellulose materials [16–18]. However, most of the previous studies have focused on the effect of LMS treatment on lignocellulose delignification and the enhancement of reducing sugar yield in the subsequent saccharification process, while key information, such as the change of biomass surface morphology and the evolution of chemical structure and composition in the pretreatment process, was rarely investigated. In addition, the enzymatic hydrolysis kinetics of wheat straw pretreated with laccase has not been proposed, which would lead to an inability to predict the lignocellulosic biomass hydrolysis process after laccase treatment. This is not advantageous for designing a bioreactor with an efficient hydrolysis process.

In the present work, effects of laccase pretreatments on physicochemical characteristics of alkali lignin and wheat straw were comprehensively investigated. Subsequently, the kinetics of enzymatic hydrolysis using pretreated wheat straw were assessed. The aims of this study were to:

- Characterize the effect of LMS pretreatment on the chemical group, the composition and the crystallinity of lignocellulosic feedstocks.
- Assess the characteristics of hydrolytic saccharification of lignocellulose after LMS pretreatment.
- Identify the adsorption characteristics and the kinetic model of enzymes on laccase-treated lignin.
- Propose an enzymatic hydrolysis kinetic model that considers adsorption of enzymes on treated lignin.

## Results and discussion

### Characterization of lignocellulosic biomass and lignin before and after pretreatment

#### Fourier transform infrared spectroscopy (FTIR) analysis

**Fig. 1:**
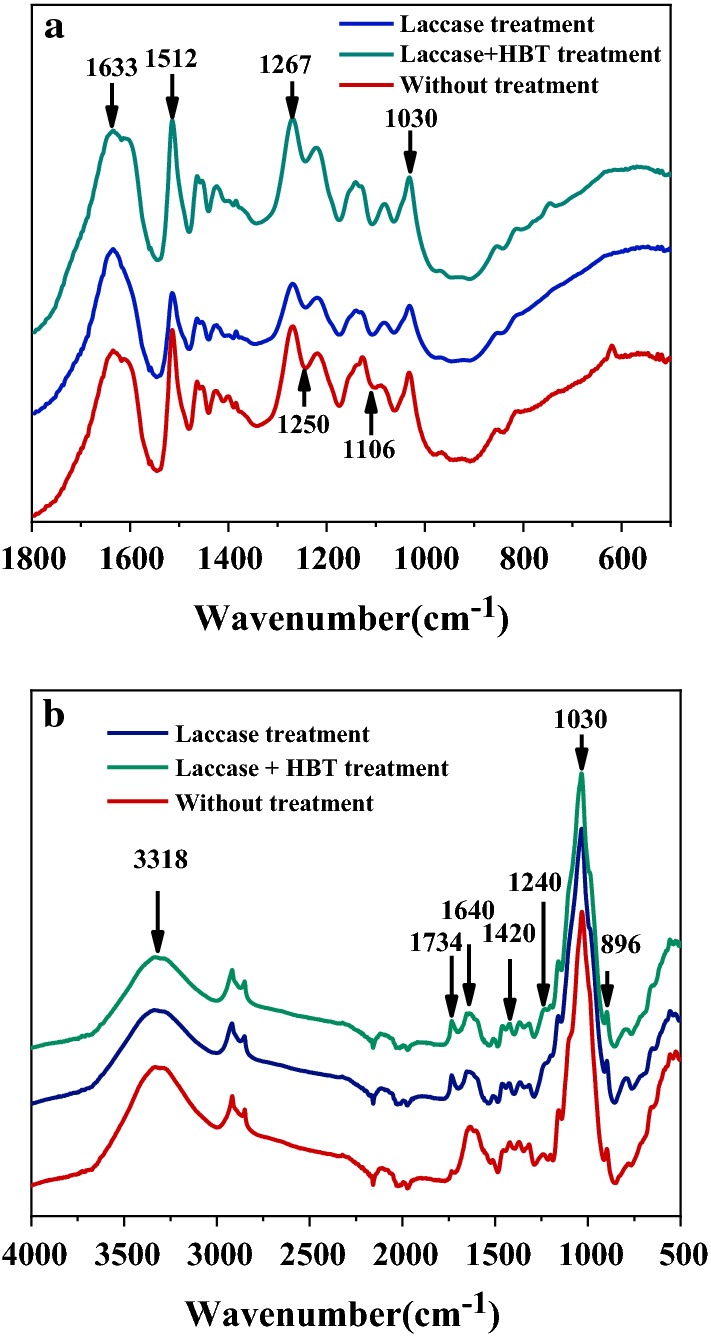
FTIR spectra of **a** alkali lignin samples before and after pretreatment, **b** lignocellulose samples before and after pretreatment

FTIR spectra of alkali lignin before and after laccase treatment are shown in Fig. 1a, and the major bands are assigned in Table 1 [19]. The fingerprint region (1600–1000 cm^−1^) in the spectra of alkali lignin significantly changed after laccase and LMS treatment, corresponding to the stretching vibrations of the different groups in lignin [20]. This suggested that the overall structure of alkali lignin had been destroyed, to some extent, after laccase and LMS pretreatment. The spectra of alkali lignin after laccase treatment shows minor changes when compared with the spectra of alkali lignin after LMS treatment. This suggested that the alkali lignin was degraded to a lesser extent when only treated with laccase. After laccase treatments, the stretching vibration peak (1106 cm^−1^) intensity of alkali lignin decreased, which was derived from an aromatic ring skeleton, indicating that some macromolecular lignin was degraded. The intensity of the peak at 1267 cm^−1^ changed slightly after laccase pretreatment, which suggested that the carbonyl in guaiacyl was ruptured during the pretreatment. The signal intensity of alkali lignin at 1633 cm^−1^ increased after LMS pretreatment, this indicated the formation of small molecule lignin in the LMS pretreatment [20].

The FTIR spectra of the untreated wheat straw, as well as the laccase-treated sample, are shown in Fig. 1b. The major bands of lignocellulosic sample are assigned in Table 1. The changes of functional groups after pretreatment were mainly concentrated in the absorption peak region (1420 cm^−1^, 1640 cm^−1^) of lignin structure characteristics, and both of them were reduced, indicating that laccase pretreatments could degrade lignin in wheat straw. The band significantly changed at 1734 cm^−1^, which is mainly attributed to the C=O vibration in acetyl and *p*-coumaroyl groups in lignin, indicating that ester bonds were cleaved in lignin [21]. The adsorption peak at 1240 cm^−1^ was from stretching vibrations of the in hemicellulose acetyl esters, which significantly changed after laccase and LMS treatment. This indicated that hemicellulose-branched chains were degraded during laccase and LMS treatment. In contrast, the intensity of the peaks at 1030 cm^−1^ and 896 cm^−1^, which corresponded to C–O stretching in cellulose and hemicellulose and C–O–C stretching at the β-glucosidic linkages in cellulose and hemicellulose respectively, did not change. This suggested the most of the polysaccharides in the lignocellulose was not degraded during the laccase pretreatment.

**Table 1:** Assignments of FTIR peaks in lignin and lignocellulose samples

Wavenumber (cm^−1^)	Assignment
3318	Stretching vibration of hydroxyl group of hydrogen bond
1734	C=O stretching of unconjugated ketone, carbonyls, and ester groups
1640, 1633	C=C stretching vibration peak in benzene ring
1510	Ring stretching vibration peak of benzene ring
1420	Deformation peak of C–H within vibration plane of aromatic ring skeleton
1267	The C–O stretching of G type lignin
1250	C–O stretching vibration in phenolic hydroxyl group
1240	Stretching vibrations of the in hemicellulose acetyl esters
1106	Carbonyl stretching vibrational peaks linked to lignin and aromatic rings
1030	C–O stretching in cellulose and hemicellulose
896	C–O–C stretching at β-glucosidic linkages in cellulose and hemicellulose

#### 2D-nuclear magnetic resonance (NMR) analysis

**Fig. 2:**
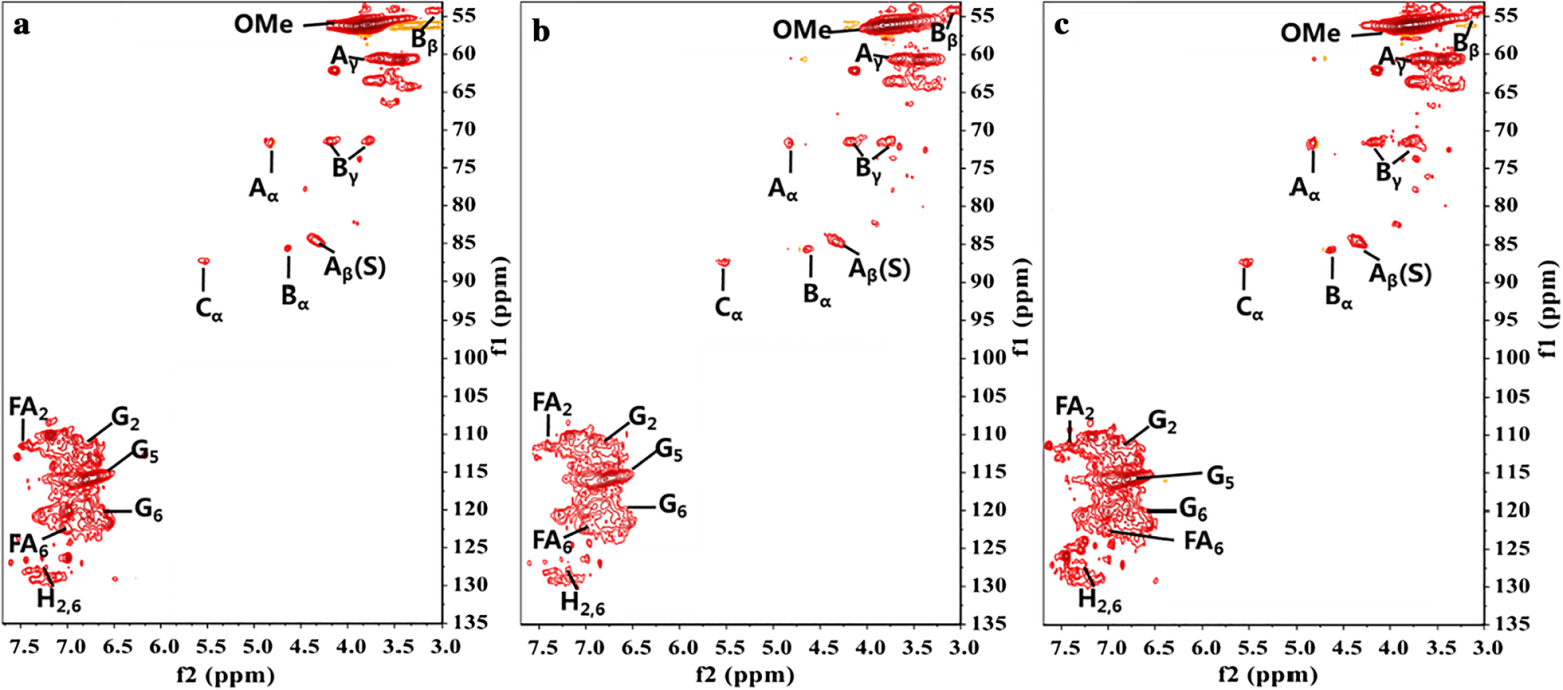
Heteronuclear single quantum correlation NMR spectra of alkali lignin samples before and after pretreatment. **a** Untreated alkali lignin, **b** laccase-pretreated alkali lignin, **c** LMS-pretreated alkali lignin

2D-NMR was used to analyze the different interunit linkages and structural units of the lignin polymers before and after laccase pretreatment (as shown in Fig. 2). The spectrum was divided into three regions: an aromatic ^13^C–^1^H correlation region (*δ*_C_*/δ*_H_ 50–90/3.0–5.5), a side chain connection ^13^C–^1^H correlation region (*δ*_*C*_*/δ*_*H*_ 100–150/6.0–8.0) and an aliphatic ^13^C–^1^H correlation region. The first two regions are often the focus of discussion because of their rich structural information. The 2D-NMR spectra of pretreated and untreated alkali lignin are assigned in Table 2 according to previous studies [17].

**Table 2:** Assignments of ^13^C–^1^H correlation signals in the HSQC spectrum of lignin samples

Label	δ_C_/δ_H_ (ppm)	Assignment
B_β_	53.4/3.11	C_β_–H_β_ in β–β (resinol) (B)
–OMe	56.2/3.78	C–H in methoxyls
A_γ_	59.5–59.7/3.25–3.73	C_γ_–H_γ_ in β-*O*-4 substructures (A)
B_γ_	71.45/4.17–3.78	C_γ_–H_γ_ in β–β resinol (B)
A_α_	71.59/4.83	C_α_–H_α_ in β-*O*-4 unit (A)
A_β_	84.78/4.32	C_β_–H_β_ in β-*O*-4′ substructures linked to a G unit (A)
B_α_	85.55/4.62	C_α_–H_α_ in β–β resinol (B)
C_α_	86.6/5.49	C_α_–H_α_ in β-5 (phenylcoumaran) substructures
G_2_	110.89/6.94	C_2_–H_2_ in guaiacyl units (G)
FA_2_	110.9/7.26	C_2_–H_2_ in FA
G_5_	115.74/6.79	C_5_–H_5_ in guaiacyl units (G)
G_6_	119.33/6.8	C_6_–H_6_ in guaiacyl units (G)
FA_6_	122.6/7.03	C_6_–H_6_ in FA
H_2,6_	127.5/7.23	C_2,6_–H_2,6_ in H units (H)

The side chain connection ^13^C–^1^H correlation region of lignin samples detailed the bond (β-*O*-4, β–β) between lignin monomers. The methoxyl groups (OCH_3_, *δ*_C_/*δ*_H_ 55.4/3.72) and β-*O*-4 aryl ether (A) are the most significant correlation signals in the side-chain region. The signals at *δ*_C_/*δ*_H_ 71.59/4.83 (A_α_), *δ*_C_/*δ*_H_ 84.78/4.32 (A_β(G)_), and *δ*_C_/*δ*_H_ 59.5–59.7/3.25–3.73 (A_γ_) belonged to the C_α_–H_α_, C_β_–H_β_, and C_γ_–H_γ_ correlations of the β-*O*-4′ ether substructures. The signal intensity at *δ*_C_*/δ*_H_ 84.78/4.32 (A_β_) showed a slight decrease after laccase and LMS pretreatment. This indicated that laccase pretreatment was effective in cleaving aromatic ether bonds in the lignin and destroying the three-dimensional network of lignin. This would increase the accessibility of cellulose to promote enzymatic hydrolysis efficiency.

The aromatic region mainly reflects the correlation signal of the aromatic ring in alkali lignin. In this region, only signals of the aromatic ring in the guaiacyl (G) and *p*-hydroxyphenyl (H) structural units can be observed, but the aromatic ring signal of the syringyl (S) structural unit is not observed. This may be due to the low content of syringyl (S) structural units in alkali lignin. The main signal peaks of the guaiacyl unit are located at *δ*_C_*/δ*_H_ 110.89/6.94, 115.74/6.79 and 119.33/6.8, which are derived from C_2_–H_2_, C_5_–H_5_ and C_6_–H_6_, respectively. Interestingly, when compared to untreated AL, the signal of the guaiacyl (G) unit in the pretreated samples showed no significant changes. This phenomenon may be due to the interference of oxidized guaiacyl units.

#### X-ray diffraction (XRD) analysis

**Fig. 3:**
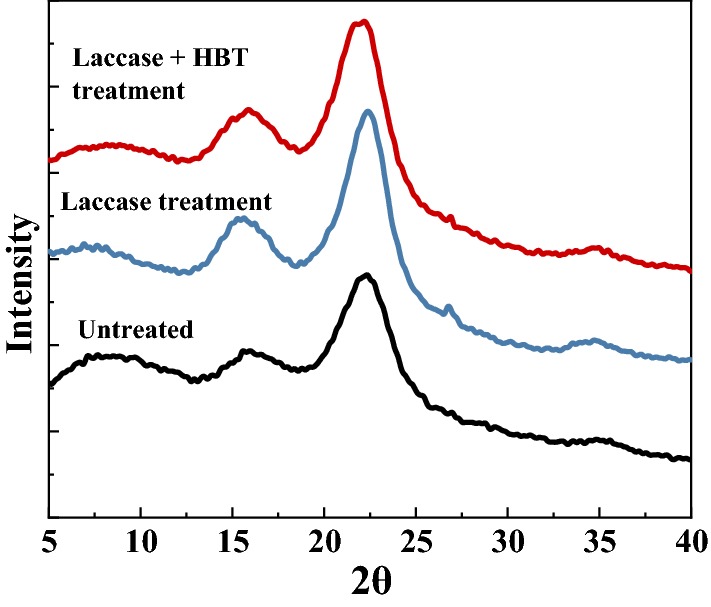
XRD spectra of lignocellulose samples before and after pretreatment

The XRD patterns of the untreated and laccase treated samples are shown in Fig. 3. The crystallinity index of pretreated wheat straw was 27% (laccase) and 28.57% (LMS), which were both higher than the untreated raw materials (26%). The partial degradation of amorphous hemicellulose and removal of lignin during pretreatment led to the increase of the proportion of crystalline cellulose. This resulted in an increase in the crystallinity of the wheat straw residue after laccase pretreatment. A slight increase in crystallinity does not increase the resistance of lignocellulose to enzymatic degradation, while the removal of lignin can reduce the nonproductive adsorption of the enzyme and increase the yield of reducing sugar. In addition, partial removal of hemicellulose and lignin can increase the accessibility of cellulose, which would also increase the yield of reducing sugar. In general, the higher crystallinity of the lignocellulose after laccase pretreatment represents a higher reducing sugar yield.

#### Elemental analysis

The element analysis and degree of unsaturation of the untreated and laccase-treated samples are shown in Table 3. The degree of unsaturation of lignocellulose was further reduced by LMS pretreatment, which indicated that the dense structure of lignocellulose was depolymerized during pretreatment, and some of the macromolecular lignin was degraded. The sulfur content of each sample decreased after laccase treatment, which may be due to the dissolution of sulfur in the pretreatment process.

**Table 3:** Elemental contents of lignocellulose and lignin

Samples	C (wt%)	O (wt%)	H (wt%)	N (wt%)	S (wt%)	Formula of C_900_	Degree of unsaturation
Ligno	44.66	47.7	5.47	1.336	0.83	C_900_H_1323_O_721_N_23_S_11_	251
Ligno La	42.34	50.12	5.94	1.245	0.354	C_900_H_1515_O_799_N_23_S_3_	155
Ligno La + HBT	41.23	51.64	5.53	1.322	0.277	C_900_H_1449_O_845_N_25_S_2_	188

#### Thermogravimetric (TG) analysis

**Fig. 4:**
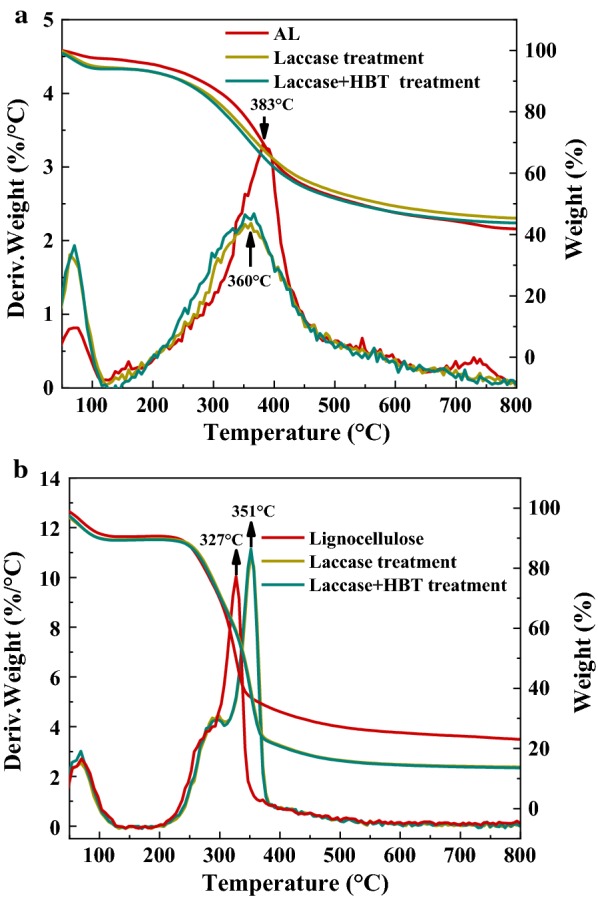
DTG and TG analysis of **a** AL before and after pretreatment, **b** lignocellulose before and after pretreatment

Figure 4a shows the TG and differential thermogravimetric (DTG) curves of untreated and laccase-treated lignin and lignocellulose samples. The pyrolysis process of the lignocellulose samples can be divided into four main stages: moisture and very light volatile components that produced during laccase pretreatment removal (< 120 °C); degradation of hemicellulose (220–315 °C); lignin and cellulose decomposition (315–400 °C) and lignin degradation (> 450 °C). However, alkali lignin only has a dehydration stage and a lignin degradation stage. As indicated in Fig. 4a, the residue char contents of untreated and laccase-treated alkali lignins were 42%, 44% and 46%, respectively. The DTG curve that corresponded to raw alkali lignin showed a maximum decomposition rate at 385 °C, but the temperature peak of alkali lignin after laccase treatments had a left shift. This suggested that the alkali lignins were converted to small molecule aromatic compounds with poor thermal stability during the laccase treatment process.

The DTG curves related to lignocellulose and treated lignocellulose are shown in Fig. 4b. The peak shoulder indicated hemicellulose decomposition, while the main peak indicated cellulose and lignin degradation. Treating with laccase and HBT-shifted hemicellulose and cellulose degradation to a higher temperature (280 °C and 351 °C, respectively). This is because part of the lignin and the hemicellulose in the wheat straw were degraded, and the removal of hemicellulose and lignin led to increased crystallinity of the raw materials, which gives a higher thermal stability [22].

The residue char contents of lignocellulose decreased significantly after laccase treatment. The residual char content is related to the ratio between the guaiacyl and the syringyl unit in the lignin [23]. The larger the proportion of the guaiacyl unit, the more char that remains. The presence of *p*-coumarates acylating the *γ*-OH of the S lignin side chains can hamper the action of laccase, therefore, laccase mainly oxidizes the G lignin in the pretreatment and leads to lower residue char content, which has been confirmed by previous 2D-NMR results.

### The adsorption characteristics and the kinetics model of cocktail enzyme on lignin

#### Effect of lignin modification on enzymatic hydrolysis of Avicel

**Fig. 5:**
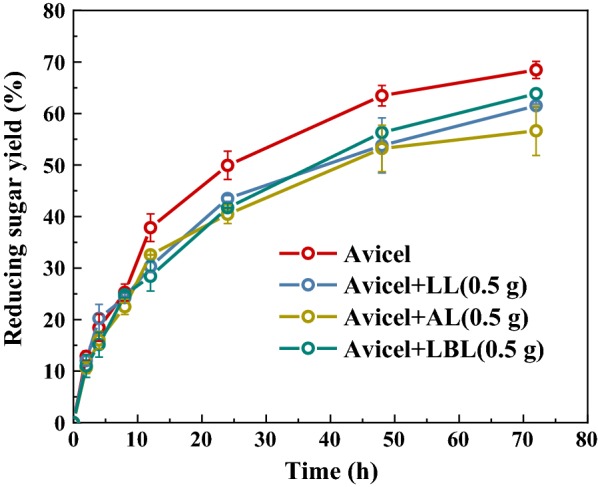
Effects of modified lignins on glucose yield in enzyme saccharification of Avicel

Figure 5 presents the enzymatic hydrolysis of Avicel with or without the addition of lignins. All lignins had an inhibitory effect on reducing sugar yield. The 72 h glucan conversion efficiencies with addition of AL (alkali lignin), LL (laccase treated alkali lignin), and LBL (LMS treated alkali lignin) were 53%, 58%, and 60%, respectively. The Avicel without lignin addition had a maximum glucose yield at 72 h of 65%. Although the addition of all lignins had an inhibitory effect on the hydrolysis saccharification process of Avicel, the modified lignins had a less negative effect than untreated alkali lignin. It is due to the adsorption capacity of alkali lignin on hydrolysis enzyme decreased after laccase treatment.

#### Effects of alkali lignin modification on nonproductive adsorption of enzyme

**Fig. 6:**
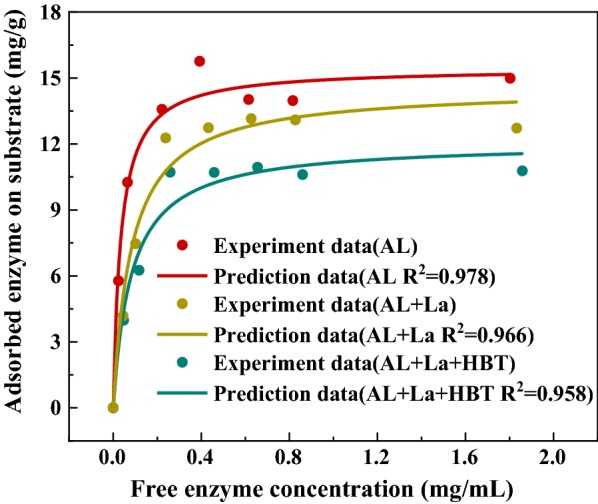
Cellulase enzyme adsorption on alkali lignin before and after pretreatment

Adsorption isotherms were generated using untreated and laccase treated lignins, and they were incubated with different enzyme loadings at 50 °C for 3 h (Fig. 6). Table 4 shows the adsorption parameters, which were estimated by fitting the enzymatic adsorption data to the Langmuir model. The maximum adsorption capacities (*E*_max_) of lignins (AL, LL, LBL) were 15.45, 14.54 and 12.07 mg/g lignin, respectively. Alkali lignin had the strongest adsorption capacity, and could adsorb, approximately, 20–28% more protein than modified lignins. This is consistent with the results of previous hydrolysis experiments. This may be due to the decrease in polymerization degree of alkali lignin after laccase treatment, which generates more exposed carboxylic groups. This can make the lignin more hydrophilic, which decrease the hydrophobic interaction between lignin and enzyme, thus decreasing the nonproductive binding for cellulase and hemicellulase. Compared with laccase treated with alkali lignin alone, the adsorption capacity of lignin can be reduced by the interaction of the mediator (HBT) and laccase, which is due to the addition of the mediator promoting the alkali lignin to degrade to aromatic compounds with less condensation.

**Table 4:** Langmuir adsorption isotherm parameters

Substrate	*E*_max_ (mg/g)	*K* (mL/mg)
AL	15.45	28.55
AL + La	14.54	11.97
AL + La + HBT	12.07	12.11

As seen in Table 4, the AL had the highest affinity (*K*) for enzyme (28.55 mL/mg protein), while LL and LBL had lower affinity for enzyme (11.97 mL/mg protein and 12.11 mL/mg protein). The strength of interaction between lignin and enzyme was characterized by the coefficient (*K*_p_), which was calculated using the maximum adsorption capacity multiplied by the Langmuir adsorption constant. The AL had the strongest binding with enzymes, which was about 3 times higher than those of the modified lignins. The results confirmed adsorption capacity of lignin was significantly reduced after lignin modification via LMS treatment, which would be very beneficial for subsequent enzymatic hydrolysis.

#### The enzymatic hydrolysis of laccase-treated lignocellulose

**Fig. 7:**
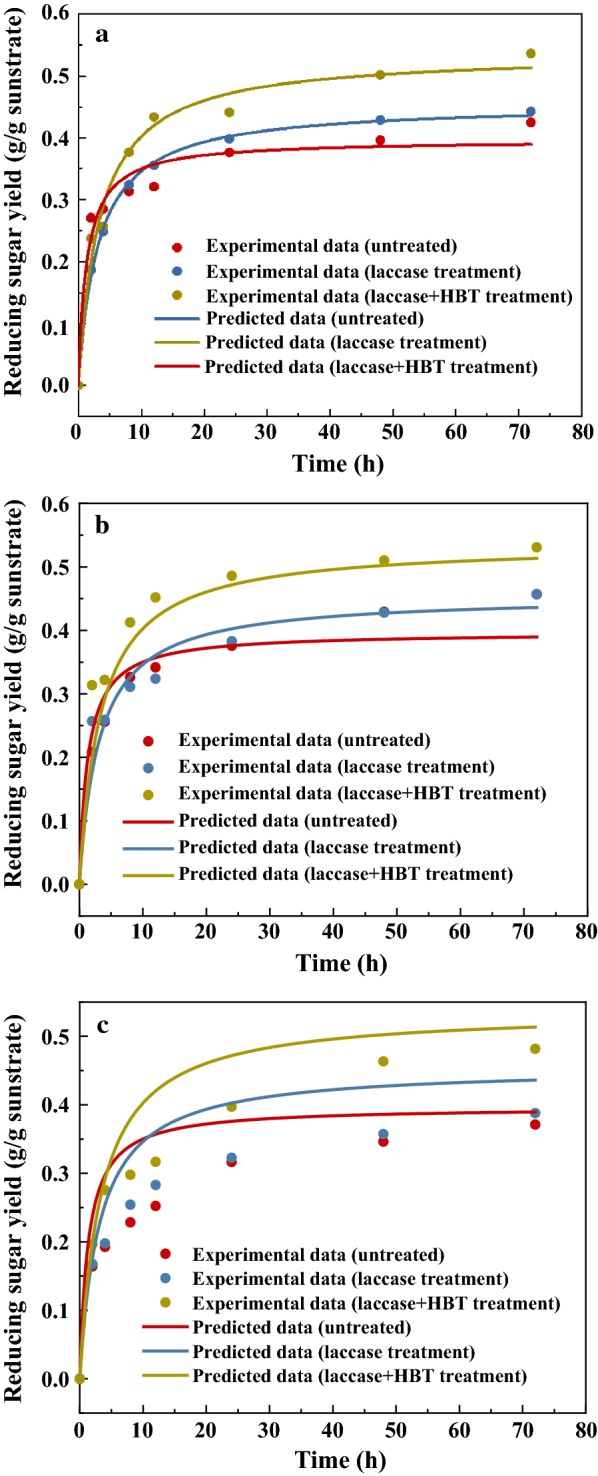
Enzymatic hydrolysis of lignocellulose **a** (5% solid loading) before and after pretreatment, **b** (2.5% solid loading) before and after pretreatment, **c** (7.5% solid loading) before and after pretreatment

Figure 7a shows the reducing sugar yield of treated and untreated lignocellulose in the enzymatic saccharification process. Compared with the raw wheat straw, laccase pretreatment and LMS pretreatment effectively improved the reducing sugar yield in the enzymatic saccharification process. The maximum reducing sugar yield of 375.9 mg/g wheat straw was obtained with the LMS pretreatment, which increased by 26% compared to the raw material. These results suggested that LMS pretreatment can increase the accessibility of cellulose and reduce the unproductive adsorption of lignin on hydrolytic enzymes, which is consistent with the previous characterization. Whereas, laccase pretreated lignocellulose had a lower reducing sugar yield than LMS-pretreated lignocellulose in the enzymatic saccharification process. This indicates that the LMS pretreatment degrades more non-phenolic lignin than the laccase pretreatment, which causes less lignin inhibition on the enzymatic hydrolysis.

**Fig. 8:**
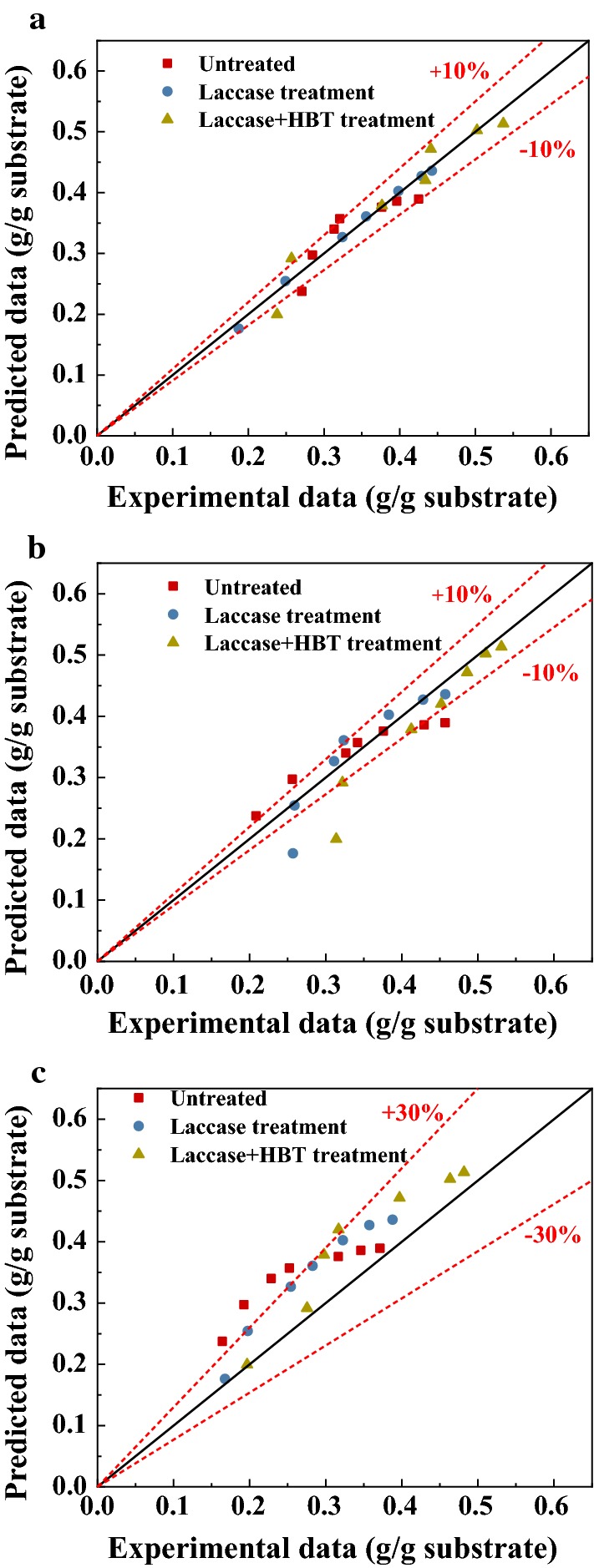
Comparison of kinetic model predictions with experimental data **a** 5% solid loading, **b** 2.5% solid loading, **c** 7.5% solid loading

The values of *K*_*M*_ and *α* can be determined from the experimental data using nonlinear regression, which is shown in Table 5, and the high *R*^2^ values (0.962–0.998) showed good fit for the experimental results using the present model, with the predicting error of kinetic model generally less than 10% (Fig. 8a). *K*_*M*_ represents the initial rate of enzymatic hydrolysis, and *α* is the time-dependent decay coefficient that represents the effect of deactivation of the enzyme. Compared with laccase-treated lignocellulose, the untreated lignocellulose had the highest initial reaction rate. This is due to the degradation of lignin in the pretreatment process, which may expose the crystalline cellulose, increasing the difficulty of cellulose degradation by cellulase. This is consistent with the increase of crystallinity of lignocellulose after laccase treatment. Another cause of the degradation, is that a small portion of the easily hydrolyzed hemicellulose in laccase treatment is degraded, which results in the initial hemicellulose hydrolysis rate being relatively slow.

**Table 5:** Enzymatic hydrolysis kinetic parameters

Substrate	*K*_*m*_ (h^−1^)	*α* (h^−1^)	*R*^2^ of fitting
Untreated	0.2935	0.598	0.962
Laccase pretreatment	0.14263	0.2437	0.998
LMS pretreatment	0.157	0.214	0.980

#### Application of the kinetic model to various substrate concentrations

To verify the applicability of the model to other hydrolysis conditions, experiments were conducted to investigate the effect of different solid loading on the enzymatic hydrolysis of laccase-pretreated and LMS-pretreated lignocellulose. Experimental data of reducing sugar yield were used to validate the proposed enzymatic kinetic model. The fitting results of 2.5% solid loading are shown in Fig. 7b, and the predicting error of kinetic model of 2.5% solid loading is shown in Fig. 8b. The predicting error of kinetic model error is generally less than 10%, which suggested that the advanced M–M model showed good prediction for LMS-pretreated lignocellulose. However, the untreated lignocellulose had a slightly higher 72 h reducing yield than the predicted model, which suggested that viscosity is an important factor affecting the hydrolysis reaction of lignocellulose feedstock. The lower the solid concentration of the reaction system, the lower the viscosity, which leads to a higher mass transfer performance, and benefits for the enzymatic hydrolysis of lignocellulose [24].

As shown in Fig. 7c, when the solid content is 7.5%, the prediction values were higher than the experimental values in most cases, and the effect of prediction became worse with increasing time. This may be due to the high viscosity that increased mass transfer resistance during the diffusion of cellulase and hemicellulase to unhydrolyzed lignocellulose, thereby affecting the rate of carbohydrate hydrolysis. As shown in Fig. 8c, the predicting error of kinetic model of treated lignocellulose is less than 30%. On the contrary, the predicting error of kinetic model of untreated lignocellulose exceeded 30% in many cases. It indicated that the mass transfer had less inhibition on the lignocellulose hydrolysis process of the LMS-pretreated sample than the untreated sample. This may be because the hydrolysis system after LMS pretreatment has a higher free enzyme concentration than the untreated, which would weaken the effects of mass transfer limitations on hydrolysis. The initial mixture viscosity of 7.5% solid loading is higher than the 2.5% solid loading of the LMS-pretreated lignocellulose, and it was also found that the 2.5% solid loading had a higher initial hydrolysis rate than the 7.5% solid loading. In summary, different solids loading systems have various prediction accuracy. To be better suited to a variety of different reaction conditions, the kinetic model can be modified by adding parameters that affect the mass transfer characteristics of the enzyme to substrate, such as solids and reactor size, in further study. The limitation of the model might be that it can only be applied in a solid–liquid system with non-immobilized enzymes, and a more accurate prediction can be attained when more time-dependent data of the yields are provided.

## Conclusion

The effect of LMS treatment on the adsorption ability of the enzyme to alkali lignin has been investigated. Alkali lignin showed the strongest adsorption capacity, which can adsorb approximately 20–28% more protein than modified lignins. Physiochemical analyses showed that the chemical groups of lignin and lignocellulose were changed, with the crystallinity of the lignocellulose increasing after LMS treatment. The reducing sugar yield during subsequent enzymatic hydrolysis increased by 26% after LMS treatment. A semi-empirical kinetic model was proposed to estimate the reducing sugar yield, and it showed high accuracy (predicting error < 10%) for describing the behavior of laccase treated wheat straw hydrolysis in the low solid loading range. This study provides a prediction approach of the enzymatic hydrolysis of biomass pretreated by laccase, which would be beneficial for optimizing the enzymatic hydrolysis process and for designing the bioreactor.

## Methods

### Materials

The wheat straw used in the experiment was obtained from the Henan province, China. The dried materials (8.7% moisture) were ground in a grinder and screened with a 40 mesh screen. The composition of the dried wheat straw was 47.97% cellulose, 20.31% hemicellulose, 21.18% lignin and 10.54% ash. The alkali lignin was purchased from Sigma-Aldrich. All samples were stored in a dryer at room temperature for composition analysis and further use.

#### Laccase pretreatment

The treatment using laccase from *Trametes versicolor* and HBT as redox mediator was employed to degrade or modify the lignin in the wheat straw and the alkali lignin. The treatment trials were carried out in 100 mL pressurized reactors. 2 g (dry weight) of the sample were added to the reactors at 5% solid loading (w/w) in 0.1 M sodium citrate buffer (pH 4.8) under O_2_ atmosphere (2 bar) and then placed inside a shaker bath maintained at 170 rpm and 50 °C for 24 h [17].

#### Enzymatic hydrolysis of Avicel

Enzymatic hydrolysis of Avicel was conducted in a flask containing sodium citrate buffer (0.1 M, pH 4.8) for 72 h at 50 °C. Specifically, the solid substrate loading rate was 5% (w/w), and the mixture was stirred in a shake bath at 170 rpm. The hydrolysis enzyme used in the experiment was a commercial complex enzyme called Cellic CTec2 (Novozymes, China) with an activity of 200 FPU/mL. The dose of hydrolysis enzyme (SAE0020) was 50 FPU/g substrate. The reducing sugar yield in the hydrolysis process was determined by the DNS method [25].

#### Enzyme adsorption on lignin

The adsorption isotherm of enzyme on the alkali lignin, before and after laccase treatment, was studied with different the enzyme protein concentrations, which ranged from 0.1 to 2.0 mg/mL [26]. Alkali lignin and the enzyme were incubated in a flask that contained 0.1 M sodium citrate buffer (pH 4.8) at 50 °C with shaking (170 rpm) for 3 h to reach equilibrium. The free protein concentration was determined using the Bio-Rad protein assay, which is a Bradford-based colorimetric method, and BSA (Sigma-Aldrich) was used as a standard [26].

#### Enzymatic saccharification of wheat straw

The enzymatic saccharification of untreated or pretreated wheat straw was carried out using the Cellic CTec2. The enzyme loading used in the saccharification process was 50 FPU/g. The hydrolysis was conducted in a 100 mL flask that contained 38 mL of sodium citrate buffer (0.1 M, pH 4.8) for 72 h at 50 °C. Specifically, the solid substrate loading ratio was 5% (w/w), and the mixture was stirred in a shake bath at 170 rpm. The reducing sugar yield in the hydrolysis process was determined by the DNS method.

#### Chemical structural analysis of the alkali lignin and lignocellulose

The determination of the chemical composition of raw and pretreated wheat straw was performed according to the National Renewable Energy Laboratory (NREL) [27]. First, 300 mg of wheat straw was mixed with 3 mL of 72% (w/w) H_2_SO_4_ at 30 °C for 60 min in a water bath, and then the reaction mixture was diluted to 4% (w/w) with 84 mL of deionized water and autoclaved at 121 °C for 60 min. The lignin content was determined by solid residue, and cellulose and hemicellulose content were determined from monosaccharides in the filtrate using HPLC (High Performance Liquid Chromatography).

FTIR spectroscopic analysis was assessed using a Thermo Scientific Nicolet iN10 FTIR Microscope (Thermo Nicolet Corporation) equipped with a DTGS detector. Scans were conducted at 400–4000 cm^−1^. Background scanning was performed for correction before data collection. The X-ray diffraction (XRD) method was used to determine the crystallinity of the samples by using an X-ray diffractometer (PANalytical Empyrean, Netherlands). The crystallinity index (CrI) was calculated as expressed in Eq. (1).

1 $${\text{CrI}}\,{ = }\,\frac{{I_{002} \, - \,I_{\text{am}} }}{{I_{002} }}\, \times \,100\%$$

*I*_002_ is the intensity of the (002) peak at approximately 2*θ *=22.5°, and *I*_am_ is the peak intensity of the amorphous region at approximately 2*θ *=18.3° [28].

The HSQC NMR spectra of lignins were detected at 25 °C using a Bruker AVIII 500 MHz spectrometer (Bruker Biospin, Fallanden, Switzerland). The sample (50 mg) was dissolved in 0.5 mL dimethyl sulfoxide-d_6_ and then transferred into an NMR tube. All configurations were referenced to the previous literature [29]. The thermogravimetric properties of the samples were analyzed via an STA409PC TG analyzer (NETZSCH, Germany) at a constant heating rate of 5 °C/min. The samples were placed in the oven to dry at 105 °C for 2–3 h to remove moisture from the samples before TG analysis. A Vario Macro cube element analyzer (Elementar, Germany) was used to determine the content of C, H, N and S in the samples. The content of oxygen was calculated as 100% minus the content of the other elements.

#### Kinetics and mathematical modeling

##### Adsorption kinetics model

According to the published literature [30], the adsorption of enzyme on lignin can be described well by the following Langmuir equation:

2 $$\left[ {E_{\text{ads}} } \right] = \frac{{\left[ {E_{ \text{max} } } \right] \cdot K \cdot \left[ {E_{\text{free}} } \right]}}{{1 + K \cdot \left[ {E_{\text{free}} } \right]}}$$

*E*_ads_ is the amount of enzyme adsorbed on the lignin (mg/g lignin), *E*_free_ is the free enzyme concentration (mg/mL) in the suspension, *E*_max_ is the maximum enzyme adsorption capacity of lignin (mg/g lignin), and *K* is the Langmuir adsorption constant (mL/mg enzyme).

##### Enzymatic hydrolysis kinetic model

Enzymatic hydrolysis of wheat straw was conducted in a heterogeneous system. A kinetics model of the enzymatic hydrolysis process was established on the basis of the Michaelis–Menten equation. The advanced M–M model assumes that (1) the adsorption of enzymes on the solid substrate is very fast in comparison with the enzymatic reactions; (2) the effect of deactivation of the enzyme is considered as a time-dependent decay coefficient; (3) *w* represents the mass ratio of lignin to cellulose and hemicellulose, and it was determined as 3:7 according to the components analysis in this study; (4) the cellulase and hemicellulase enzymes were assumed to form a combination that can degrade carbohydrates in the lignocellulose to produce reducing sugar; (5) [*ES*] is at a quasi-steady-state. The advanced M–M model is illustrated by the scheme:

3 $$E{\mkern 1mu} + {\mkern 1mu} S\underset{{k_{{ - 1}} }}{\overset{{k_{1} }}{\longleftrightarrow}}ES\xrightarrow{{k_{2} }}E + P$$

S, E, P, and ES represent the substrate (polysaccharide), the complex of free enzyme, and the product, respectively. The reaction rate constants, *k*_1_ and *k*_2_, are the forward reaction rates. The reaction rate constant *k*_−1_ represents the reverse reaction rate. The enzymatic hydrolysis of wheat straw can be expressed by the equation:

4 $$\frac{{d\left[ {ES} \right]}}{{{\text{d}}t}} = k_{1} \left[ E \right]\left[ S \right] - {k_{ - 1}} \left[ {ES} \right] - k_{2} \left[ {ES} \right] \cong 0$$

5 $$\left[ {ES} \right] = \frac{{k_{1} \left[ E \right]\left[ S \right]}}{{k_{ - 1} + k_{2} }}$$

6 $$\frac{d\left[ P \right]}{{{\text{d}}t}} = k_{2} \left[ {ES} \right] = \frac{{k_{1} k_{2} }}{{k_{ - 1} + k_{2} }}\left[ E \right]\left[ S \right]$$

The following equation is based on the fundamental principles of mass conservation:

7 $$\left[ {S_{0} } \right] = \left[ S \right] + \left[ {ES} \right] + \lambda \left[ P \right]$$

[*S*_0_] represents the initial concentration of the substrate, the constant *λ* represents the average conversion coefficient of cellulose and hemicellulose to reducing sugar, and the value was set as 0.9 for all calculations. Because of the M–M model assumption that $\left[ {ES} \right]\, \ll \,\left[ P \right]\, + \,\left[ S \right]$, [*ES*] can be negligible, therefore, Eq. (4) can be simplified as:

8 $$\left[ {S_{0} } \right] = \left[ S \right] + \lambda \left[ P \right]$$

After the integration of Eqs. (2)–(8), the dependence of the product concentration by time was obtained:

9 $$\frac{d\left[ P \right]}{{{\text{d}}t}}\, = \,\frac{{k_{1} k_{2} }}{{k_{ - 1} \, + \,k_{2} }}\left[ E \right]\left( {\left[ {S_{0} } \right]\, - \,\lambda \left[ P \right]} \right)$$

Taking the mass balance of the enzyme gives,

10 $$\left[ {E_{0} } \right]\, = \,\left[ E \right]\, + \,\left[ {ES} \right]\, + \,\left[ {E_{ad} } \right]\, + \,\left[ {E_{\text{ina}} } \right]$$

[*E*_0_] is the initial concentration of the enzyme, [*E*_ad_] is the concentration of the enzyme adsorbed on the lignin, and [*E*_ina_] is the concentration of the inactivated enzyme. Because the effect of deactivation of the enzyme is considered to be a time-dependent decay coefficient (*α*), the equation for enzyme adsorption and inactivation can be expressed as [31]:

11 $$\left[ {E_{\text{ad}} } \right] = w\left[ {S_{0} } \right]\left[ {E_{\text{ads}} } \right]$$

12 $$\frac{{d[E_{\text{ina}} ]}}{{{\text{d}}t}} = k_{\text{ina}} [E]$$

13 $$k_{\text{ina}} \, = \,\frac{2\alpha }{1\, + \,\alpha t}$$

Combining Eqs. (11)–(13) gives

14 $$\frac{\left[ P \right]}{{\left[ {S_{0} } \right]}} = {{\left( {1 - \exp \left( {\frac{{ - \lambda K_{m} t}}{1 + \alpha t}} \right)} \right)} \mathord{\left/ {\vphantom {{\left( {1 - \exp \left( {\frac{{ - \lambda K_{m} t}}{1 + \alpha t}} \right)} \right)} \lambda }} \right. \kern-0pt} \lambda }$$

15 $$K_{m} = \frac{{k_{1} k_{2} }}{{k_{ - 1} + k_{2} }}\left( {\left[ {E_{0} } \right] - w\left[ {S_{0} } \right]\left[ {E_{\text{ads}} } \right]} \right)$$

*K*_*m*_ is the initial observed reaction rate, and it can be concluded from the equation that the initial reaction rate is related to the initial enzyme concentration and the enzyme adsorbed on lignin. *K*_*m*_ and *α* were determined from the experimental data using a nonlinear regression by Origin software.

## Data Availability

All data generated or analyzed during this study are included in this article.

## Abbreviations

LMS: laccase-mediated system
HBT: 1-hydroxybenzotriazole
FTIR: Fourier transform infrared spectroscopy
2D-NMR: 2D-nuclear magnetic resonance
XRD: X-ray diffraction
FPU: filter-paper unit
TG: thermogravimetric
DTG: differential thermogravimetric
AL: alkali lignin
LL: laccase-treated lignin
LBL: laccase- and HBT-treated lignin
S: syringyl
G: guaiacyl
H: *p*-hydroxy phenylpropane
